# Arginine-Specific Mono ADP-Ribosylation *In Vitro* of Antimicrobial Peptides by ADP-Ribosylating Toxins

**DOI:** 10.1371/journal.pone.0041417

**Published:** 2012-08-07

**Authors:** Marta Castagnini, Monica Picchianti, Eleonora Talluri, Massimiliano Biagini, Mariangela Del Vecchio, Paolo Di Procolo, Nathalie Norais, Vincenzo Nardi-Dei, Enrico Balducci

**Affiliations:** 1 Novartis Vaccines & Diagnostics, Siena, Italy; 2 School of Biosciences and Biotechnologies, University of Camerino, Camerino, Italy; 3 Department of Evolutionary Biology, University of Siena, Siena, Italy; Institute Pasteur, France

## Abstract

Among the several toxins used by pathogenic bacteria to target eukaryotic host cells, proteins that exert ADP-ribosylation activity represent a large and studied family of dangerous and potentially lethal toxins. These proteins alter cell physiology catalyzing the transfer of the ADP-ribose unit from NAD to cellular proteins involved in key metabolic pathways. In the present study, we tested the capability of four of these toxins, to ADP-ribosylate α- and β- defensins. Cholera toxin (CT) from *Vibrio cholerae* and heat labile enterotoxin (LT) from *Escherichia coli* both modified the human α-defensin (HNP-1) and β- defensin-1 (HBD1), as efficiently as the mammalian mono-ADP-ribosyltransferase-1. *Pseudomonas aeruginosa* exoenzyme S was inactive on both HNP-1 and HBD1. *Neisseria meningitidis* NarE poorly recognized HNP-1 as a substrate but it was completely inactive on HBD1. On the other hand, HNP-1 strongly influenced NarE inhibiting its transferase activity while enhancing auto-ADP-ribosylation. We conclude that only some arginine-specific ADP-ribosylating toxins recognize defensins as substrates *in vitro*. Modifications that alter the biological activities of antimicrobial peptides may be relevant for the innate immune response. In particular, ADP-ribosylation of antimicrobial peptides may represent a novel escape mechanism adopted by pathogens to facilitate colonization of host tissues.

## Introduction

Human defensins are cationic multifunctional arginine-rich peptides (molecular masses ranging from 3.5 to 6 kDa) characterized by three intramolecular disulfide bridges that stabilize their structure [1]–[4]. Defensins display microbicidal activity against a wide spectrum of Gram-negative and Gram-positive bacteria, fungi and viruses [5]. They are also cytotoxic for epithelial cells and chemotactic for T-cells. Based on the presence of six conserved cysteine residues and sequence homology, human defensins are grouped into α- and β- defensins. The first group (α-defensins) includes human neutrophil peptides (HNP)-1 to 4, major components of the azurophilic granules of neutrophils, and two enteric human defensins, HD-5 and HD-6, isolated from the granules of Paneth cells in the small intestine, [6]. The second group (β-defensins), is mainly expressed in epithelial cells of various organs [7]–[9]. It has been shown that ADP-ribosylation of HNP-1 on arginine 14 reduces its antimicrobial and cytotoxic activities [10]. Mono ADP-ribosylation consists in the enzymatic transfer of the single ADP-ribose moiety of NAD to specific amino-acid residues of acceptor proteins coupled to the release of nicotinamide (nam) [11]. In mammals this reaction is catalyzed by a family of ADP-ribosyltransferases (ART1-5) [12], [13], while the best studied ADP-ribosylation reactions are those catalyzed by bacterial ADP-ribosylating toxins. The ADP-ribosylation of a large panel of host proteins catalyzed by bacterial toxins leads to the interruption of cellular metabolic and regulatory pathways causing severe diseases [14]. *Vibrio cholerae* toxin (CT) [15], *Escherichia coli* heat labile enterotoxin (LT) [16], *Pseudomonas aeruginosa* exoenzyme S (ExoS) [17] and the recently discovered NarE, a toxin-like protein from *Neisseria meningitidis* [18], recognize arginine as an ADP-ribose acceptor in a similar fashion to ART1 and ART5 [13], [19]. Arginine specificity is conferred to ARTs by the presence of the R-S-EXE triad signature in the active site [20]. Recent studies indicated that α-defensins display a novel biological function consisting in the ability to neutralize the activity of potent bacterial toxins like lethal factor, a metalloprotease produced by *Bacillus anthracis* [21], and toxin B produced by *Clostridium difficile* [22]. Moreover it has been shown that HNP1-3 neutralize the cytotoxic effects exerted by diphtheria toxin (DT) and *Pseudomonas aeruginosa* exotoxin A (ETA), while they were inactive on CT and pertussis toxin (PT) [23]. The neutralization of toxins with selected amino-acid specificity prompted us to hypothesize that mono ADP-ribosylation of specific amino-acids may block defensin ability to inhibit the activities of toxins. Therefore, we evaluated whether HNP-1 could be recognized by arginine-specific bacterial ARTs. In the present paper we provide evidence that CT and LT ADP-ribosylated α- and β- defensins, which thus represent novel substrates for these bacterial ARTs. On the other hand, NarE and ExoS did not modify either α- or β- defensins. Interestingly, unmodified HNP-1 exerted inhibition on NarE transferase activity suggesting a regulatory role. While the ADP-ribosyltransferase activity was inhibited by HNP-1, the NAD-glycohydrolase (NADase) activity remained unaltered. Furthermore, HNP-1 strongly enhanced the auto-ADP-ribosylation of NarE, a recently discovered catalytic activity of this toxin. Overall, our data highlight the interplay between ADP-ribosylating toxins and human defensins.

## Results

**Figure 1:**
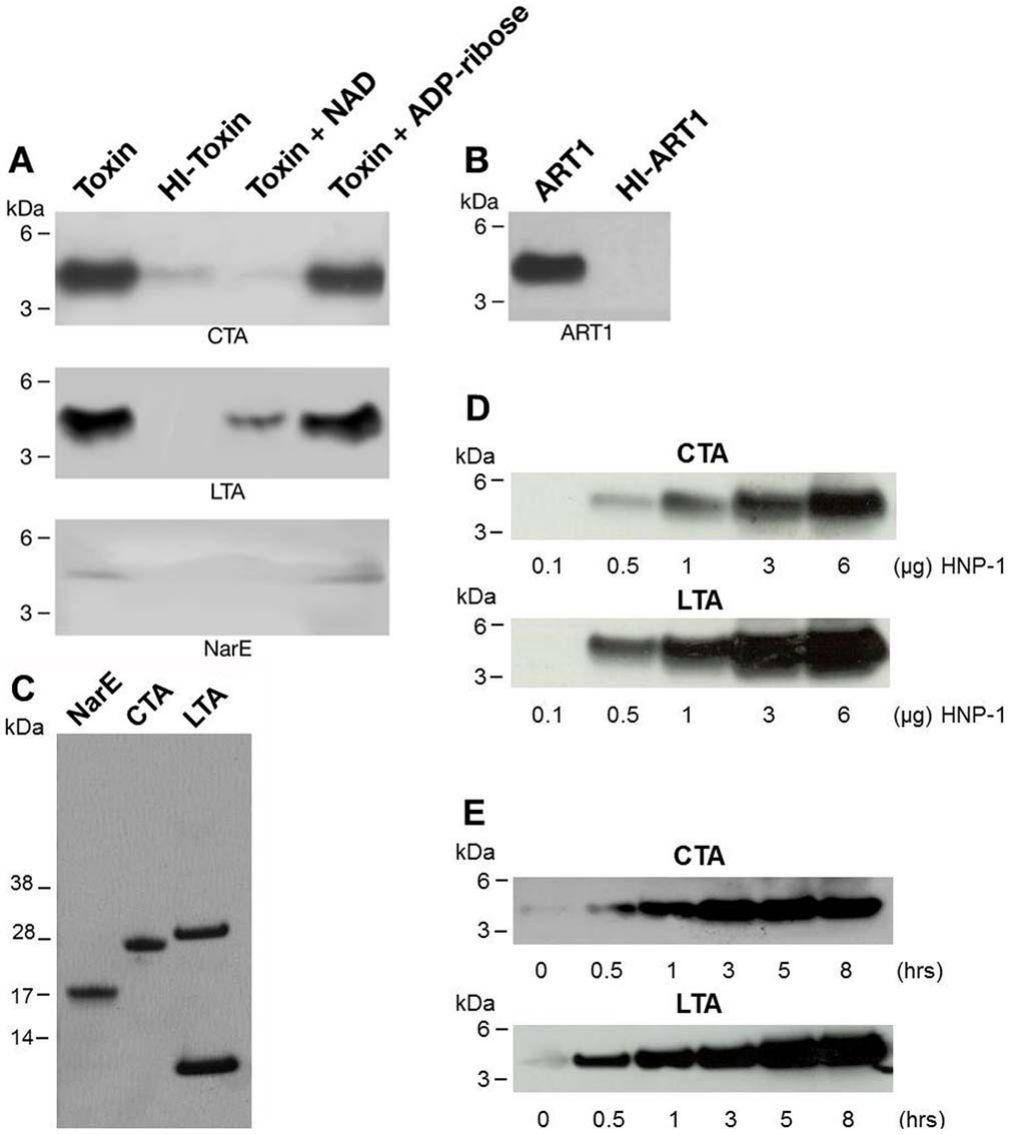
Modification of HNP-1 by selected ADP-ribosyltransferases. (**A**) HNP-1 is ADP-ribosylated by CTA and LTA but only weakly by NarE. HNP-1 (3 µg, 43.56 µM) was incubated with CTA (2.5 U), LTA (8.9 U) or NarE (2 U) and 10 µM of biotin-NAD in 50 mM potassium phosphate buffer, pH 7.5, at 30°C for 1 h (Toxin). The same reactions were performed with heat-inactivated toxins (HI-Toxin), in the presence of 2 mM NAD (Toxin + NAD), or 2 mM ADP-ribose (Toxin + ADP-ribose). The ADP-ribosylated peptides were resolved by SDS-PAGE in a 10% NuPAGE gel, using MES as running buffer and transferred to nitrocellulose. After blocking with 5% BSA in PBS containing 0.05% Tween-20 (PBS-T) for 1 h, the blot was incubated with streptavidin-HRP conjugated (1∶10000 dilution) for 1 h at RT in the same buffer. The biotin-ADP-ribose labeled bands were visualized by chemiluminescence. (**B**) ART1 ADP-ribosylated HNP-1. HNP-1 (3 µg, 43.56 µM) was incubated with ART1 (6.8 U) and 10 µM of biotin-NAD in 50 mM potassium phosphate, pH 7.5 at 30°C for 1 h (ART1). A control reaction with heat-inactivated ART1 is also shown (HI-ART1). (**C**) SDS-PAGE analysis of the purification grade of 2 µg each of CTA, LTA and NarE. (**D**) HNP-1 is ADP-ribosylated in a dose and response dependent manner by CTA and LTA. HNP-1 at the concentration shown in the Figure was incubated with CTA (2.5 U), or LTA (8.9 U) and 10 µM of biotin-NAD in 50 mM potassium phosphate buffer, pH 7.5, at 30°C for 1 h. (**E**) HNP-1 is ADP-ribosylated in time dependent fashion. HNP-1 (3 µg) was incubated with CTA (1.25 U) or LTA (4.45 U) using the same conditions above described for the times of incubation indicated in the Figure. Molecular markers are on the left. Data shown are representative of several experiments performed in the same conditions.

**Figure 2:**
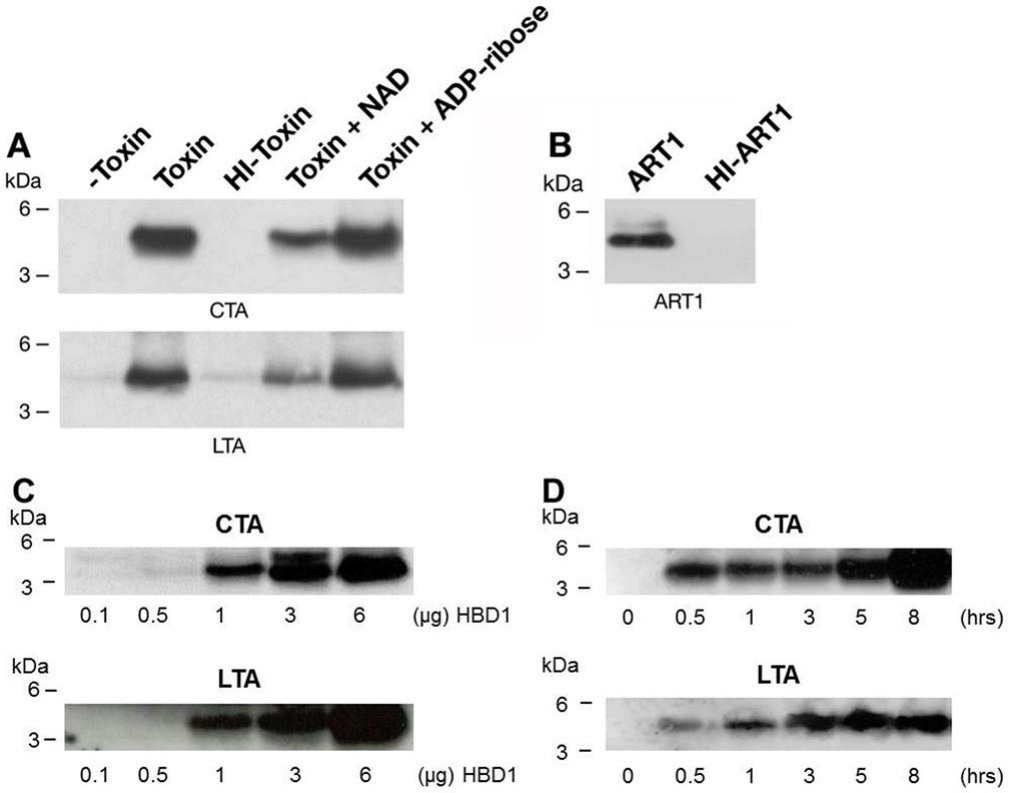
Modification of β**-defensin by selected ADP-ribosyltransferases.** (**A**) HBD1 is ADP-ribosylated by CTA and LTA. HBD1 (3 µg, 38.18 µM)) was incubated with CTA (2.5 U) or LTA (8.9 U) in the presence of 10 µM biotin-NAD, in 50 mM potassium phosphate buffer, pH 7.5 at 30°C for 1 h (Toxin). Reactions were also performed in the presence of 2 mM NAD (Toxin + NAD) or 2 mM ADP-ribose (Toxin + ADP-ribose). Control reactions performed with heat-inactivated CTA or LTA (HI-Toxin) or in the absence of toxins (-Toxin) are also shown. The ADP-ribosylated peptides were separated by SDS-PAGE in a 10% NuPAGE gel and transferred to nitrocellulose. The membrane was treated as previously described, incubated with streptavidin-HRP conjugated (1∶10000 dilution) before visualization of the biotin-ADP-ribose labeled bands by chemiluminescence. (**B**) ADP-ribosylation of HBD1 by ART1. HBD1 (3 µg, 38.18 µM) was incubated with 6.8 U of ART1 (ART1) or heat-inactivated ART1 (HI-ART1) and 10 µM biotin-NAD in 50 mM potassium phosphate buffer, pH 7.5, at 30°C for 1 h. (**C**) HBD1 is ADP-ribosylated in a dose response fashion. HBD1 at the concentration shown in the Figure was incubated with CTA (2.5 U) or LTA (8.9 U) in the presence of 10 µM biotin-NAD, in 50 mM potassium phosphate buffer, pH 7.5 at 30°C for 1 h. (**D**) Time dependent ADP-ribosylation of HBD1. HBD1 (3 µg) was incubated with CTA (1.25 U) or LTA (4.45 U) using the same conditions above described. Times of incubation are indicated in the Figure. Molecular markers are on the left. Data shown are representative of two independent experiments.

**Figure 3:**
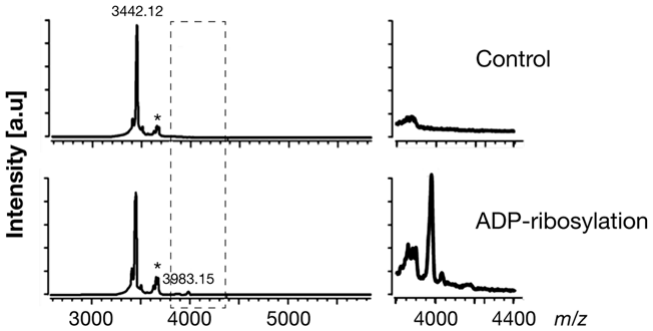
MALDI-TOF mass spectra of HNP-1 reaction with CTA. Mass spectra analysis confirmed the mono-ADP-ribosylation of HNP-1 by CTA after incubation at 30°C for 1h in the presence of 2 mM NAD. Upper panels (left side: spectrum of *m/z* 2500 – 5300, right side: zoomed spectrum of *m/z* 3800 – 4300) show the mass of the control reaction, i.e. HNP-1 incubated only with NAD without toxin (*m/z* 3442.12). Lower panels (left side: spectrum of *m/z* 2500 – 5300, right side: zoomed spectrum of *m/z* 3800–4300) represent the unmodified HNP-1 peptide and the product of ADP-ribosylation peptide by CTA (*m/z* 3983.15). Stars (*) correspond to Sinapinic Acid adducts (+206 Da).

**Figure 4:**
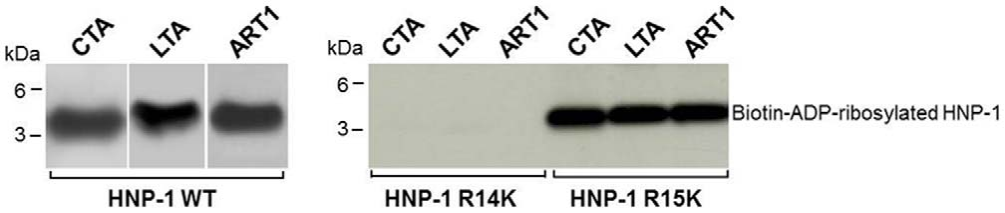
HNP-1 is ADP-ribosylated at R14. HNP-1 R14K and HNP-1 R15K protein variants (3 µg, 43.85 µM) were incubated with CTA (2.5 U), LTA (8.9 U) or ART1 (5.5 U) in the presence of 10 µM biotin-NAD, in 50 mM potassium phosphate buffer, pH 7.5 at 30°C for 1 h. The ADP-ribosylated peptides were separated by SDS-PAGE in a 10% NuPAGE gel and transferred to nitrocellulose. Membranes treated as previously described, were incubated with streptavidin-HRP conjugated (1∶10000 dilution) before visualization of the biotin-ADP-ribose labeled bands by chemiluminescence. Here shown in comparison with the modification of HNP-1 wild-type in the same reaction conditions.

**Figure 5:**
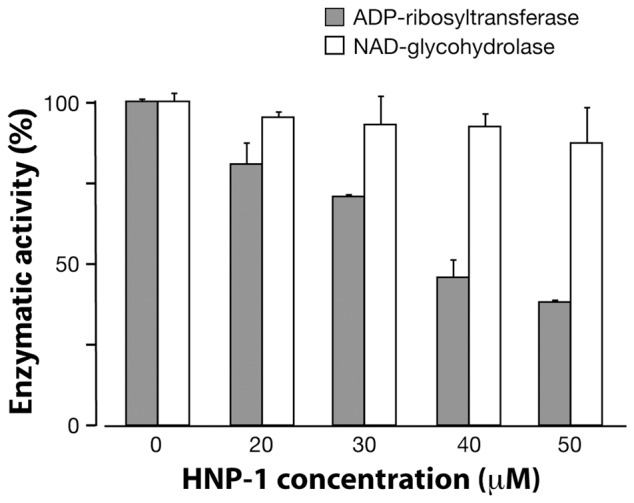
HNP-1 inhibited the ADP-ribosyltransferase, but not the NAD-glycohydrolase activity of NarE. NarE (20 µg) was incubated with 0.1 mM NAD [*adenine*-U-^14^C]NAD (0.05 µCi), in 50 mM potassium phosphate buffer, pH 7.5, 20 mM agmatine as an ADP-ribose acceptor at 30°C for 18 h in the presence of the indicated concentrations of HNP-1. The synthesized ADP-ribosylagmatine was purified through Dowex AG 1-X2 and the incorporated radioactivity was eluted in water and counted in a counter (grey bars). To monitor the NADase activity (white bars), similar reactions in the absence of agmatine and with 0.1 mM [*carbonyl*-^14^C]NAD (0.05 µCi) replacing [*adenine*-U-^14^C]NAD (0.05 µCi) were performed. The radioactive nam was eluted in water in Dowex AG 1-X2 and counted. Values represent the means ± S.D. of two independent assays.

**Figure 6:**
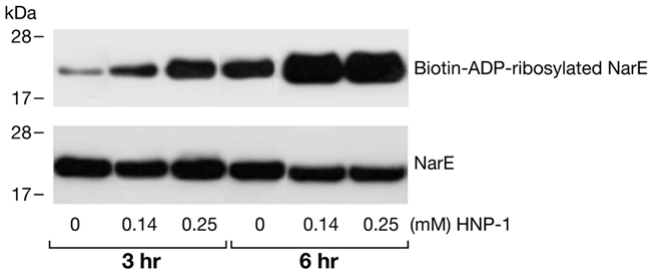
HNP-1 enhanced the auto-ADP-ribosylation of NarE. Purified NarE (0.4 µg) was auto-ADP-ribosylated with 10 µM biotin-NAD in 50 mM potassium phosphate pH 7.5 at 30°C for 3 and 6 h in the presence of the indicated concentrations of HNP-1. Proteins were separated by SDS-PAGE and transferred to a nitrocellulose membrane. To detect biotin-ADP-ribosylated NarE, the membrane was incubated with streptavidin-HRP (upper panel). For western blotting the nitrocellulose membrane was incubated with rabbit polyclonal α-NarE (1∶10000 dilution) and with α-rabbit HRP-conjugated (lower panel). Labeled bands were detected by chemioluminescence. Data shown are representative of two independent experiments.

To establish whether arginine-specific bacterial ARTs can ADP-ribosylate HNP-1, we incubated HNP-1 with the catalytic A subunit of CT (CTA), LT (LTA), ExoS or NarE individually. As shown in Fig 1A, CTA and LTA catalyzed the transfer of the biotin-ADP-ribose from biotin-NAD to HNP-1 with an efficiency that was comparable to that of ART1 (Fig. 1 B). This incorporation was strongly reduced after heat-inactivation of the toxins (Fig. 1 A). CTA and LTA have both transferase, and NADase activity [24], [16]. The latter produces ADP-ribose that can react non-enzymatically with lysine residues in proteins [25]. However, since the incorporation of biotin-ADP-ribose on HNP-1 was strongly reduced in the presence of 2 mM unlabelled NAD (200-fold excess) but not with 2 mM ADP-ribose, we could rule out that the reaction was non-enzymatic. The enzymatic nature of the reaction was further confirmed in the dose dependent (Fig. 1 D) and time-course experiments (Fig. 1 E), showing that the increase of modified peptide is dependent on the level of free substrate and by the incubation time. In this respect the purification grade of the toxins (Fig. 1 C) is shown, to exclude the possibility of a blockage of the peptide by contaminating proteins. Under the same conditions, HNP-1 was a poor substrate for NarE (Fig. 1 A) compared to ART1 (Fig. 1 B). ExoS was completely inactive towards HNP-1 (data not shown), in agreement with a previous report [26]. ADP-ribosylation of antimicrobials by CT and LT is not restricted to HNP-1. Also HBD1, which contains only one arginine at position 29 and is constitutively expressed by epithelial cells in the airway [27], was ADP-ribosylated (Fig. 2 A). As for HNP-1, labelling did not occur in the presence of heat-inactivated toxins. The addition of an excess of unlabelled NAD to the reaction mixture decreased the incorporation of an ADP-ribose moiety on HBD1, while the incorporation of biotin-ADP-ribose on HBD1 was not reduced by the presence of 2 mM ADP-ribose. Dose-dependent reactions and time course experiments support the enzymatic nature of the modification also in the case of HBD1 (Fig. 2 C, D). NarE and ExoS did not modify HBD1 (data not shown). In contrast with a previous report [26], HBD1 was modified by ART1 to the same extent of HNP-1 (Fig. 2 B). To confirm that the observed modifications corresponded to the addition of the ADP-ribose unit, the products of the reaction of CTA with HNP-1 in the presence of NAD were identified by MALDI-TOF MS. As shown in Fig. 3, these included a peptide of 3442.12 Da, consistent with unmodified HNP-1 (theoretical mass: 3442.1 Da) and a peptide of 3983.15 Da. Although the amount of the modified peptide was low, we can conclude that the reaction is specific since we observed a mass increase consistent with mono ADP-ribosylated HNP-1 (theoretical mass: 3983.1 Da). Similar results were obtained with the LT catalyzed reaction (data not shown). To identify the preferred arginine residue of HNP-1 modified by CTA and LTA, we used two variants of HNP-1 in which a lysine replaced the arginines at positions 14 (HNP-1-R14K) or 15 (HNP-1-R15K). We found that CTA and LTA selectively ADP-ribosylated HNP-1 at R14 (Fig. 4). Recent studies have shown that when HNP-1 is not recognized as a substrate, it is able to inhibit the ART activity of bacterial toxins such as ETA and DT [23] and also the eukaryotic ART5 multiple catalytic activities [26]. Therefore, since HNP-1 is only weakly modified by NarE, we investigated whether HNP-1 exerts a similar effect on NarE activities. The addition of HNP-1 to the reaction mixture seems to reduce the ADP-ribosyltransferase activity in a concentration dependent fashion (Fig. 5, grey bars) while the NADase activity was not greatly affected (Fig. 5, white bars). In contrast, HNP-1 enhanced the auto-ADP-ribosylation of NarE (Fig. 6 upper panel), a recently discovered activity of this toxin (Picchianti et al. manuscript in preparation).

## Discussion

ADP-ribosylating toxins are usually secreted by bacterial pathogens in the host environment. Some of them, which possess arginine-specificity, could recognize arginine-rich peptides such as α- and β- defensins as substrates. Both α- and β- defensins are released by neutrophils and epithelial cells respectively in high amounts at inflammatory sites. In this report we present evidence that synthetic HNP-1 and HBD1 are ADP-ribosylated *in vitro* by CTA and LTA. In contrast they are not recognized as substrates by ExoS and only poorly by NarE, suggesting specificity for both bacterial toxins and substrates. The artificial kemptide (PKA peptide substrate), which contains a di-arginine motif, was modified by CT on the first arginine of the motif while a mammalian ART recognized the second arginine within the R-R motif [28], [29]. In contrast, our data indicate R14 as the preferred modification site, since the HNP-1-R14K was not ADP-ribosylated by the ADP-ribosylating toxins used in this study. Our findings are in contrast with studies performed by other groups, which failed to show toxin-catalyzed incorporation of the ADP-ribose unit on defensins [23], [26]. Others evaluated the presence of the ADP-ribosylated-HNP-1 by monitoring the absorbance of the modified peptide in reversed-phase chromatography, but not being successful in identifying it [23], [26]. Therefore we chose a chemiluminescence assay to detect ADP-ribosylation because of the higher sensitivity, allowing the detection of small amounts of modified HNP-1.

In agreement with previous findings we did not observe incorporation of ADP-ribose with PT [23] and ExoS [26]. Labelling of proteins can also result from the covalent non-catalyzed reaction of NAD [30] or free ADP-ribose with the ε-amino groups of lysines [25]. ADP-ribosylation of HNP-1, in which lysine residues are absent (Table 1), was not blocked by the addition of free ADP-ribose, while a reduction of incorporation was noticed when unlabelled NAD was added to the reaction mixture. Comparable results were obtained with HBD1 that contains four lysine residues. These data, further supported by mass spectrometry analysis, strongly indicate that an enzymatic ADP-ribosylation, and not a secondary reaction with NAD or ADP-ribose, was responsible for the modification. Defensins belonging to α- and β- group contain several conserved arginines (Table 1), which are recognized by CT and LT. However they are devoid of diphtamide and asparagine residues, which are the target amino-acid of DT, ETA and clostridial toxins. Furthermore, cysteines, present in α- and β- defensins and recognized by PT as ADP-ribose acceptors, are engaged in disulphide bridges. It is well known that defensins have a variety of activities, but the antimicrobial function is by far the most important. Thus ADP-ribosylation of selected arginines might well correlate with recent discoveries, which show that antibacterial activity strictly depends on cationicity [31] and that only selective arginines support this activity [32]. Interestingly, toxic activities are not decreased in the case of CT [23], which ADP-ribosylates HNP-1 at an arginine residue. By contrast, HNP-1, which is devoid of the target amino-acids is not modified by ETA and DT, thus protecting cells from DT- or ETA-mediated cell death [23]. Besides its antimicrobial activity, several lines of evidence suggest an additional regulatory role for HNP-1 when it is not recognized as a substrate, as described for ART5 and ART1 [26]. Here we showed that HNP-1 is able to reduce NarE transferase activity. This reduction is more evident at high concentrations of HNP-1, likely to be present in the site of inflammation. Of note the NADase activity, a reaction not usually involved in the toxicity process, is not affected. On the other hand, auto-ADP-ribosylation, which could be an intramolecular mechanism regulating the two activities (Picchianti et al. manuscript in preparation) is enhanced. Although physiological substrates for CT and LT are well known and the extent of modification is limited, members of the antimicrobial peptide family may serve as novel substrates for these ADP-ribosylating toxins. At the onset of infection, bacterial pathogens have evolved different countermeasures to limit the effectiveness of antimicrobials [33] and to counteract the immune system. The modification of antimicrobial components of the innate immune system by bacterial ADP-ribosylating toxins may represent a mechanism that could facilitate bacterial colonization. In the context of the primary immune response, the ADP-ribosylation of HNP-1 may affect both the anti-microbial weapons released by neutrophils and the interplay between inflammatory cells, eventually facilitating the onset of an infectious disease. Hence, our study showing arginine-specific ADP-ribosylation of human defensins catalyzed by some bacterial toxins may be relevant in the onset of infectious diseases.

**Table 1:** Human defensins.

Human defensins		
α	DEF1_HUMAN Neutrophil defensin 1	ACYC**R**IPACIAGE**RR**YGTCIYQG**R**LWAFCC
	DEF2_HUMAN Neutrophil defensin 2	CYC**R**IPACIAGE**RR**YGTCIYQG**R**LWAFCC
	DEF3_HUMAN Neutrophil defensin 3	DCYC**R**IPACIAGE**RR**YGTCIYQG**R**LWAFCC
	DEF4_HUMAN Neutrophil defensin 4	VCSC**R**LVFC**RR**TEL**R**VGNCLIGGVSFTYCCT**R**VD
	DEF5_HUMAN Neutrophil defensin 5	ATCYC**R**TG**R**CAT**R**ESLSGVCEISG**R**LY**R**LCC**R**
	DEF6_HUMAN Neutrophil defensin 6	TCHC**RR**SCYSTEYSYGTCTVMGINH**R**FCCL
β	DEFB1_HUMAN β-defensin 1	DHYNCVSSGGQCLYSACPIFTKIQGTCY**R**GKAKCCK
	DEFB2_HUMAN β-defensin 2	GIGDPVTCLKSGAICHPVFCP**RR**YKQIGTCGLPGTKCCKKP
	DEFB3_HUMAN β-defensin 3	GIINTLQKYYC**R**V**R**GG**R**CAVLSCLPKEEQIGKCST**R**G**R**KCC**RR**KK
	DEFB4_HUMAN β-defensin 4	ELD**R**ICGYGTA**R**C**R**KKC**R**SQEY**R**IG**R**CPNTYACCL**R**K

## Materials and Methods

### Reagents

[*adenine*-U-^14^C]NAD (274 mCi/mmol) and [*carbonyl*-^14^C]NAD (53 mCi/mmol) were purchased from Amersham (Glattbrugg, Switzerland); Dowex AG 1-X2, the Bradford reagent for protein quantification and the immunoblotting detection system were purchased from Bio-Rad (Hercules, CA), while standard bovine serum albumin (BSA) was obtained from Pierce (Rockford, IL). Isopropyl-1-thio-β-D-galactopyranoside was purchased from Calbiochem (Darmstadt, Germany). SimplyBlue SafeStain was ordered from Invitrogen (Carlsbad, CA), 6-biotin-17-NAD from Trevigen (Gaithersburg, MD), synthetic defensins from Bachem (Weil am Rheim, Germany) and streptavidin HRP-conjugate from SouthernBiothech (Birmingham, AL). All other reagents used in this study were from Sigma Aldrich (Saint Louis, MO).

### Preparation of ART1 and bacterial toxins

Chinese hamster fibroblast V79 cells transfected with pTet-ON-ART1 cDNA were kindly provided by Dr. J. Mac Dermot [34]. Human ART1 was synthesized as a GPI-linked protein on the surface of V79 cells after induction for 48 h with 2 µg/ml final concentration of doxycicline. The soluble form of ART1 was collected following treatment of intact V79 cells with 1 U/ml of phosphatidylinositol-specific phospholipase C for 1 hr at 37°C [34]. NarE was produced as previously described [18]. Briefly, the *NarE* gene cloned in the pET21b+ expression vector with a carboxyl-terminal 6× histidine tag was used to transform *E. coli* BL21 (DE3) competent cells (Invitrogen). Transformed cells were grown overnight in LB broth at 37°C with gentle shaking (180 rpm) and protein synthesis occurred after addition of 1 mM isopropyl-1-thio-β-D-galactopyranoside (IPTG) for 3 h at 25°C. Following induction, bacteria were harvested, centrifuged and lysed in B-PER (bacterial-protein extraction reagent, Pierce) in the presence of Mg^++^, DNase, lysozyme and phenyl-methyl sulfonyl fluoride (PMSF) as a protease inhibitor. After centrifugation to discard debris and membranes, the soluble fraction was loaded on a Ni-NTA (Pharmacia, Biotech, Stockholm, Sweden) affinity resin and the protein was eluted according to manufacturer's instructions. CTA was purchased from Sigma Aldrich, while LTA was cloned and purified by Drs. Paolo Ruggiero and Laura Pancotto (Novartis Vaccines & Diagnostics, Siena Italy). Since all of the above toxins need sulphydril agents like dithiothreitol (DTT) to exert full activity, we performed their activation as previously described [35], [36]. Since DTT causes reduction of disulphide bridges causing linearization of HNP-1 and facilitating access of ADP-ribose to arginine acceptors, the activation mixture was extensively dialyzed in the same buffer lacking DTT using Microcon centrifugal filter devices (Amicon, Houston, TX). *Pseudomonas aeruginosa* exoenzyme S ADP-ribosyltransferase (transferase domain) and its activator FAS (Factor activating exoenzyme S) were kindly provided by Dr. Joseph Barbieri (Medical College of Wisconsin, Milwaukee WI).

### ADP-ribosyltransferase enzymatic assay

Some of the ADP-ribosylating enzymes used in this study ART1, CTA, LTA, and NarE were tested by monitoring the transfer of ADP-ribose to agmatine using a standard assay [37]. The assay was carried out in a final volume of 0.3 ml containing 50 mM potassium phosphate, pH 7.5, 20 mM agmatine and 0.1 mM [*adenine*-U-^14^C]NAD (0.05 µCi). After incubation at 30°C, duplicate samples (100 µl each) were applied to 1 ml columns of Dowex AG 1-X2. [*adenine-* ^14^C]ADP-ribosylagmatine was eluted for radio assay with 5 ml of H_2_O and the radioactivity counted in a Packard mod counter. The ART1 fraction exerted an activity of 6.8 nmol/h (U), while the activated CTA and LTA showed values of 2.5 nmol/h (U) and 1.8 nmol/h (U) respectively. NarE 0.9 nmol/h, Uwas used shortly after purification. Specific activities of the toxins used were 2.5 nmol/mg/h for CTA, 6.25 nmol/mg/h for LTA and 0.4 nmol/mg/h for NarE. ExoS activity was monitored using an auto-ADP-ribosylation assay in the presence of FAS ligand [38].

### NAD-glycohydrolase assay

An analogous assay based on nam release was employed to measure the NADase activity of NarE [24]. This assay was carried out in the same conditions described above for the transferase assay with 0.1 mM [*carbonyl*-^14^C]NAD (0.05 µCi) replacing [*adenine*-U-^14^C]NAD and without agmatine as ADP-ribose acceptor.

### Detection of biotinylated ADP-ribose-HNP-1 and immunoblot analysis

HNP-1 (3 µg, 43,56 µM) and HBD1 (3 µg, 38.18 µM) were ADP-ribosylated for 1 hour at 30°C in 50 mM potassium phosphate buffer (pH 7.4), with ART1, CTA, LTA, or NarE and 10 µM of biotinylated-NAD (biotin-NAD) in a final volume of 20 µl. In control experiments 2 mM NAD or 2 mM ADP-ribose were added individually to each assay mixture. In negative controls the ADP-ribosylating toxin was omitted from the reaction mixture or heat-inactivated. The ADP-ribosylated peptides were resolved by SDS-PAGE in a 10% NuPAGE gel, using MES as running buffer and transferred to nitrocellulose using a dry system apparatus (I-Blot, Invitrogen). The membrane was blocked for 1 h with 5% BSA in PBS containing 0.05% Tween-20 (PBS-T), extensively washed and then incubated in the same buffer containing streptavidin-HRP conjugated (1∶10000 dilution) and mixed for 1 h on a nutator at room temperature (RT). After several washings with PBS-T, bound streptavidin was detected using an ECL immunoblotting detection system (Bio-Rad) according to the manufacturer's instructions. Molecular masses were estimated from the calibration standard included in each gel.

### Mass-Spectrometry analysis of ADP-ribosylated-HNP-1

Peptide molecular masses were determined using a MALDI-TOF/TOF mass spectrometer UltraFlex (Bruker Daltonics, Bremen, GmbH). Ions generated by laser desorption at 337 nm (N_2_ laser) were recorded at an acceleration voltage of 20 kV in linear mode. In general, about 200 single spectra were accumulated to improve the signal/noise ratio and analyzed by FlexAnalysis version 2.4 (Bruker Daltonics). Briefly, 1 µl of reaction solution (20–60 pmoles) was added to 1 µl of a saturated solution of sinapinic acid (3,5-dimethoxy-4-hydroxy-trans-cinnamic acid) in 30% (vol/vol) acetonitrile, 0.1% (vol/vol) trifluoroacetic acid (TFA). Then 2 µl of peptide/matrix mixture was spotted on a stainless steel sample target and air-dried at room temperature. Peptide mass spectra were calibrated using external peptide calibration standard (Bruker Daltonics).

### Protein assay

Protein content was determined by the Bradford protein assay kit (Bio-Rad) using BSA for standardization.
